# Genome-wide association studies provide genetic insights into natural variation of seed-size-related traits in mungbean

**DOI:** 10.3389/fpls.2022.997988

**Published:** 2022-10-13

**Authors:** Jinyang Liu, Yun Lin, Jingbin Chen, Qiang Yan, Chenchen Xue, Ranran Wu, Xin Chen, Xingxing Yuan

**Affiliations:** Institute of Industrial Crops, Jiangsu Academy of Agricultural Sciences/Jiangsu Key Laboratory for Horticultural Crop Genetic Improvement, Nanjing, China

**Keywords:** multiple genome-wide association studies, QTN-by-environment interactions, *VrEmp24/25*, multi-omics analysis, RT-qPCR

## Abstract

Although mungbean (*Vigna radiata* (L.) R. Wilczek) is an important legume crop, its seed yield is relatively low. To address this issue, here 196 accessions with 3,607,508 SNP markers were used to identify quantitative trait nucleotides (QTNs), QTN-by-environment interactions (QEIs), and their candidate genes for seed length (SL), seed width, and 100-seed weight (HSW) in two environments. As a result, 98 QTNs and 20 QEIs were identified using 3VmrMLM, while 95, >10,000, and 15 QTNs were identified using EMMAX, GEMMA, and CMLM, respectively. Among 809 genes around these QTNs, 12 were homologous to known seed-development genes in rice and *Arabidopsis thaliana*, in which 10, 2, 1, and 0 genes were found, respectively, by the above four methods to be associated with the three traits, such as *VrEmp24/25* for SL and *VrKIX8* for HSW. Eight of the 12 genes were significantly differentially expressed between two large-seed and two small-seed accessions, and *VrKIX8*, *VrPAT14*, *VrEmp24/25*, *VrIAR1*, *VrBEE3*, *VrSUC4*, and *Vrflo2* were further verified by RT-qPCR. Among 65 genes around these QEIs, *VrFATB*, *VrGSO1*, *VrLACS2*, and *VrPAT14* were homologous to known seed-development genes in *A. thaliana*, although new experiments are necessary to explore these novel GEI-trait associations. In addition, 54 genes were identified in comparative genomics analysis to be associated with seed development pathway, in which *VrKIX8*, *VrABA2*, *VrABI5*, *VrSHB1*, and *VrIKU2* were also identified in genome-wide association studies. This result provided a reliable approach for identifying seed-size-related genes in mungbean and a solid foundation for further molecular biology research on seed-size-related genes.

## Background

Mungbean (*Vigna radiata* (L.) R. Wilczek) is a basic source of protein and carbohydrate, as it contains approximately 20% protein and 75% carbohydrate, and is a traditional and important legume in Asia (Somta et al., 2007). Due to its short life cycle (60–75 days), relative drought tolerance, and the ability to restore atmospheric nitrogen in association with *Rhizobium*/*Bradyrhizobium* bacteria, mungbean plays a crucial role in cropping systems and soil improvement (Somta et al., 2007; Alam et al., 2014).

The crop is generally grown as a cash crop in cereal-based farming systems. However, the major constraint in mungbean production is low seed yield. The average seed yield of mungbean is only approximately 700 kg per ha (Islam et al., 2015). Therefore, improving seed yield is the main goal in mungbean breeding. Understanding the genetic basis underlying seed-size-related traits is critical for the genetic improvement of mungbeans. In mungbeans, the ideotype of high-yielding cultivars are generally characterized by a large seed size, a short and synchronous maturity, a low sensitivity or insensitivity to day length, and the resistances to insects and disease (Fernandez et al., 1988). However, the knowledge on genes related to seed size has been limited. Moreover, the genes involved in the pathway of seed developments are not yet fully known.

Seed weight is the most important yield component and directly proportional to seed yield per plant in mungbean. To date, there have been seven studies of QTLs for seed weight in mungbean. Most of these studies are based on bi-parental segregation populations derived from interspecific crosses between cultivated and wild (*V. radiata* var. *sublobata*) mungbeans, and only two studies have evaluated seed size in more than one environments. The number of QTLs identified in those studies ranged from 3 to 11. Humphry et al. (2010) reported 11 loci for seed weight using SSR-marks, and Mei et al. (2009) identified a major QTL associated with both bruchid resistance and seed mass. Nonetheless, no candidate gene was identified for this trait.

Although many genes for seed weight have been reported in *Arabidopsis* (Plackett et al., 2012; Ge et al., 2016; Lu et al., 2016; Cheng et al., 2018; Zhang et al., 2020), soybeans, and rice (Luo et al., 2013; Ge et al., 2016; Liu et al., 2020a; Hao et al., 2021; Nguyen et al., 2021), few genes were reported in mungbean. In *Arabidopsis*, *FATB* (Bonaventure et al., 2003) was involved in the synthesis of short-chain fatty acids and influenced seed development. Although *GA20OX* regulated *Arabidopsis* in late floral development (Plackett et al., 2012), the overexpression of *GmGA20OX* in *Arabidopsis* enhanced seed size and weight. *KIX8* controlled seed size in *Arabidopsis* and soybeans (Liu et al., 2020a; Nguyen et al., 2021). *BES1* suppressed the cell elongation and increased seed size in legume species (Ge et al., 2016). *ERG2* promoted early seed development and influenced the length of mature siliques (Cheng et al., 2018). In soybeans, *GA20OX* (Lu et al., 2016), *GmFAD3* (Singh et al., 2011), *GmLEC2* (Manan et al., 2017), *GmPDAT* (Liu et al., 2020c), *GmKIX8-1* (Nguyen et al., 2021), and *GmGA3ox1* (Hu et al., 2022) were found to influence seed size by regulating lipid accumulation or increasing cell proliferation. In rice, *D1* (Sun et al., 2018), *D2* (Fang et al., 2016), *flo2* (She et al., 2010), *GS3* (Sun et al., 2018), *OsBZR1* (Liu et al., 2021), *GW2* (Hao et al., 2021), *D11* (Wu et al., 2016), and *OsHT* (Guo et al., 2020) were found to control seed weight by regulating rice grain size or starch quality.

Knowledge regarding seed development pathway is also a valuable source for transgenic strategies to improve crop production. As reported, there are several signaling pathways that control seed size, including the G-protein signaling, ubiquitin proteasome pathways, mitogen-activated protein kinase (MAPK) signaling, auxin pathways, and some transcriptional regulators (Li et al., 2019). In *Arabidopsis*, *GPA1*, *AGB*, and *AGG3* were involved in G-protein-signaling pathways. *DA1*, *DA2*, *SOD2*, *UBP15*, *EOD1*, and *SAMBA* were involved in ubiquitin proteasome pathways. In addition, *ABA2*, *ABI5*, *SHB1*, *MINI3*, *IKU2*, and *CKX* were involved in the HAIKU (IKU) pathway. Additional genes were found to be related to seed size developments, but their pathways are uncertain, such as *KIX8*, *BES1*, *MES1*, and *KLU* (Orozco-Arroyo et al., 2015; Li et al., 2019). However, some reports have been focused on genetic foundation and molecular mechanism of seed developments in mungbean.

Genome-wide association studies (GWASs), along with multi-omics analysis, have been frequently used to mine candidate genes for most important agronomic traits in crops. Integrating GWAS with comparative genomics, transcriptome analysis, and molecular experiments, genes have been identified to be associated with complex traits (Liu et al., 2020c). For example, Gong et al. (2022) conducted a GWAS with high-quality single nucleotide polymorphism (SNP) data and seed-size traits, and found that Cla97C05G104360 and Cla97C05G104380, which are involved in abscisic acid metabolism, played important role in regulating the seed size in watermelon. Duan et al. (2022) identified *GmST05* to be associated with soybean seed size through the GWAS of 1800 soybean germplasm resources, and *GmST05* differed significantly at the transcriptional level. Liu et al., 2022a,c used GWASs and biological experiments to identify a pleiotropic gene *GmPDAT* for seed size- and oil-related traits in soybean, and a salt-stress-tolerance gene *VrFRO8* in mungbean. Nonetheless, the related genes responsible for seed-size-related traits remained unknown in mungbean.

To address the above issues, 196 mungbean accessions with 3,607,508 SNP markers were used to conduct GWAS for seed length (SL), seed width (SW), 100-seed weight (HSW) using 3VmrMLM (Li et al., 2022b), efficient mixed-model association expedited (EMMAX) (Kang et al., 2010), genome-wide efficient mixed-model association (GEMMA) (Zhou and Stephens, 2012), and compressed mixed linear model (CMLM) (Zhang et al., 2010) methods. Candidate genes around quantitative trait nucleotides (QTNs) and QTN-by-environment interactions (QEIs) for the three traits were predicted by transcriptomics and comparative genomics. Key candidate genes were verified by RT-PCR analysis. Moreover, genes in seed-development-regulation pathway were also mined by comparative genomics. It should be noted that *VrEmp24/25* and *VrKIX8* were found to be associated with SL and HSW, and a major gene *VrPAT14* (LOD = 61.95, *r*^2^ = 5.80%) was identified in QEI detection *via* 3VmrMLM.

## Materials and methods

### Plant materials and treatments

A diverse set of 196 mungbean accessions including 20 wild and 176 cultivated accessions from 23 countries, were used in this study (Supplementary Data Set 1). All the accessions were planted in a randomized complete block design with two replicates in an experimental field of Kasetsart University, Kamphaeng Saen Campus, Nakhon Pathom, Thailand in 2018 and 2020. In each replicate, each accession was planted in a single row 2.5 m long with 12.5 cm intra-row spacing (ca. 20 plants/row) and 50 cm inter-row spacing. Cultural practices were performed according to Park (1978). SW (mm), SL (mm), and HSW (g) were measured. At maturity. The SL and SW traits for each accession were averaged based on 20 seeds and 100SW for each accession was averaged based on three replicates.

### Whole-genome resequencing

The young leaves of the above 196 mungbean accessions were collected 1 week after planting. The DNA was extracted in 2018, using the CTAB method (Smith et al., 2005). Short reads sequenced by an Illumina HiSeq 4000 platform (Illumina, San Diego, CA, United States), and mapped to scaffolds using Burrows-Wheeler-Alignment Tool (BWA) (Version 0.7.15)^1^ (Li and Durbin, 2009). Genome Analysis Toolkit (GATK) was used to select SNP and indel^2^ (McKenna et al., 2010). Sulv 1 genome was selected as the reference genome in the GATK analysis (Yan et al., 2020). High-quality SNPs and Indel variations were obtained as the following steps. (a) Retaining concordant sites both identified by GATK and VCFtools were retained (Danecek et al., 2011). (b) Filtering out SNP with quality value below 30, removing SNPs with an average coverage depth < 8× and with minor allele frequency (MAF) less than 5%. (c) Deleting insertions and deletions (InDels) with length less than10 bp were deleted. A total of 3,607,508 SNPs were identified.

As described in Liu et al. (2022a), the number of subpopulations was five (*K* = 5), and the population structure (Q matrix) was calculated using ADMIXTURE software (version is 1.3.0).^3^ The K matrix was calculated using the above CMLM (GAPIT version 3),^4^ EMMAX (GAPIT),^5^ GEMMA (Version 0.94.1)^6^, and 3VmrMLM programs (IIIVmrMLM)^7^ (Supplementary Data Set 2; Li et al., 2022a).

### Genome-wide association study for seed width, seed length, and 100-seed weight

Only the SNPs with MAF ≥ 0.05 and missing rate < 10% were used in GWAS (Pongpanich et al., 2010). The lines with more than 95% missing for trait were filtered out (Liaw and Wiener, 2002). SW, SL, and HSW, and the above SNP markers in 196 mungbean accessions were used to conduct GWAS using four different methods, including 3VmrMLM (Li et al., 2022b) *via* software IIIVmrMLM (Li et al., 2022a), EMMAX (Kang et al., 2010), GEMMA (Zhou and Stephens, 2012), and CMLM (Zhang et al., 2010). The probability threshold for significant QTNs was set at 1/*m* = 2.77e-07 (*m* = 3,607,508) for all the four methods (Xu et al., 2018; Zhang Y. M. et al., 2019; Zhang Y. M. et al., 2019), and the LOD score threshold for suggested QTNs was set at LOD ≥ 3.0 for 3VmrMLM (Li et al., 2022b). Heatmaps of the linkage disequilibrium was generated by LDheatmap package (Shin et al., 2006), haplotype analysis was conducted by LDheatmap package (Barrett et al., 2005). The averages for those traits measured in 2018 and 2020 were used in GWAS.

### Candidate gene identification

Candidate genes for salt tolerance were mined in the follow steps. (a) All the genes between the 30 Kb around regions for each of the significantly QTN were mined, where the LD-value was about 20 Kb in mungbean, (b) mined the *Arabidopsis*, rice and soybean homologous genes of those candidate genes, which were reported related to seed developments, seed production, phytohormone signaling pathways and carbohydrate metabolism pathways, etc. (Li et al., 2019), as the candidate genes. (c) The selected genes showing different expression between two groups of mungbean accessions contrasting in seed size (large seed vs. small seed) (see below) were considered as candidate genes.

### Differentially expressed gene based on RNA-sequenced data

Two large-seeded accessions [G141 and G143; 19.32 ± 7.09 (g)] and two small-seeded accessions [G169 and G171; 11.58 ± 5.93 (g)] were selected for RNA sequencing (RNA-seq) analysis. Data in seed set were collected at three seed development stages (10, 15, and 25 DAF) for RNA extraction in 2021. Total RNA was extracted using RNAprep Pure Plant Kit (DP441) according to the manufacturer’s instructions. 1 μg high-quality RNA samples (OD260/280 = 1.8∼2.2; OD260/230 ≥ 2.0; RIN ≥ 6.5; 28S:18S ≥ 1.0 and >10 μg) were used to construct the sequencing library (G9691B, Agilent). The RNA were analyzed in an Illumina Novaseq Sequencer. Raw reads were cleaned by trimmomatic^8^ (Bolger et al., 2014), and clean reads were mapped to reference sequences using Hisat2 (Pertea et al., 2016). The gene expression level was calculated by using RPKM method by Subread package (Mortazavi et al., 2008).

In the key candidate gene identification, the extracted RNA in two large-seeded accessions at 10 and 25 DAF were treated with RNase-free DNase I (Promega, Madison, WI, United States). After reverse transcription, the cDNA was used as a template for RT-qPCR using the Takara Bio TB Green Premix Ex Taq (Tli RNase H Plus). The detail progress was described by Liu et al. (2022b). Reactions were run on a Bio-Rad CFX96 system. *EVM0007380* (homologous of *At3g18780*) was used as the CK in this experiment. Primers were designed by NCBI and tested by RCR of tubulin. The *t*-test was adopted in the hypothesis testing, *P* < 0.05, *P* < 0.01, and *P* < 0.001 indicated significant probability levels at 0.05, 0.01, and 0.001, respectively. Information of the primers used is presented in Supplementary Table 1.

### Protein–protein interaction

The protein–protein interactions (PPIs) were detected used the online tools STRING^9^ (Jensen et al., 2009). The mungbean (*V. radiata* (L.) R. Wilczek) protein database was used as the protein library.

## Results

### Phenotypic variation for mungbean seed-size-related traits

**FIGURE 1:**
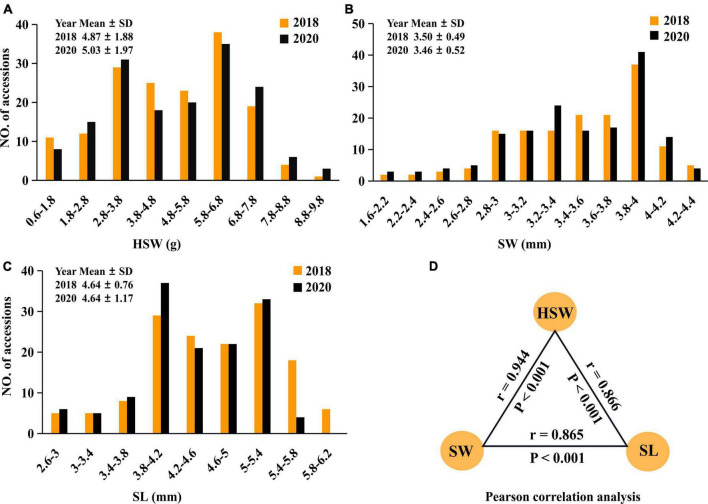
The frequency distributions of seed-size-related traits. Frequency distributions of HSW **(A)** (g), SL **(B)** (mm), and SW **(C)** (mm) in 196 mungbean accessions, which were measured in 2018 (brown bar) and 2020 (black bar). SD, standard deviation. The associations of HSW with SW and SL, the average dates of those traits measured in 2018 and 2020 were used in the partial correlation analysis **(D)**.

100-seed weight, SW, and SL in 196 mungbean accessions were measured in 2018 and 2020. The average-plus-standard deviations for the three traits across the 2 years were 5.05 ± 1.91 (g), 3.48 ± 0.51 (mm), and 4.64 ± 0.99 (mm), respectively, and their average coefficients of variation (CV) across the 2 years were 38.5, 14.5, and 16.5 (%), respectively (Supplementary Table 2). Although the trends for those traits in the 2 years were similar (Figures 1A–C), HSW (38.5%) had much larger phenotypic variation than SW (14.5%) and SL (16.5%), indicating their large phenotypic variation and typical quantitative traits. In general, the wild mungbeans showed low seed weight (1.68 ± 0.61) as well as short SW (2.45 ± 0.401) and SL (3.12 ± 0.43), while the cultivated mungbeans had high seed weights (5.29 ± 1.68) as well as long SW (3.56 ± 0.41) and SL (4.76 ± 0.92) (Supplementary Table 2). Moreover, significant difference for each trait between the 2 years was observed (*P* < 0.001), and these traits had significant correlations with each other (*r* > 0.87, *P* < 0.001) (Figure 1D), indicating the existence of common QTNs among these traits (Liu et al., 2020b).

### Genome-wide association studies for seed-size-related traits in mungbean

#### Detection of main-effect quantitative trait nucleotides for seed-size-related traits in each environment

**FIGURE 2:**
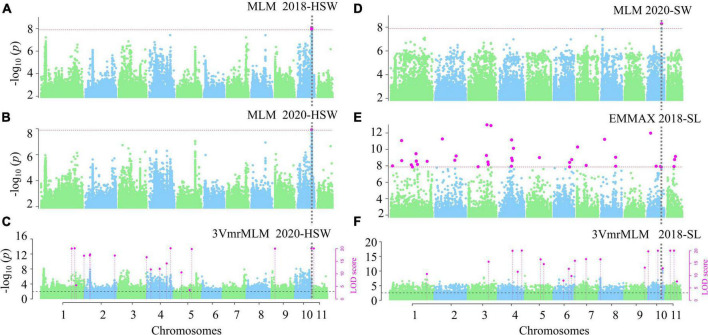
Manhattan plots for the GWAS for seed-yield-related traits. GWAS for HSW **(A–C)**, SW **(D)** and SL **(E,F)**. Significant QTN in phenotypic GWAS was set at *P*-value ≤ 0.05/m = 1.39e-08 (*m* = 3607508), ≤2.77e-09 for CMLM and EMMAX **(A,B,D,E)**; and LOD ≥ 3.0 for the 3VmrMLM as the significant QTN, and all the critical values were marked by horizontal lines, *Y*-axis on the left side reports –log10 *P*-values of SNP, while *Y*-axis on the right side reports LOD scores, and LOD scores are shown in points with straight lines.

After removing the SNPs with an average coverage depth < 8× and with a MAF less than 5%, we identified more than 3.6 million SNP markers. In the single-environment analysis, the phenotypic observations for each trait in 196 accessions measured in 2018 and 2020 were used to associate with 3,607,508 SNPs using 3VmrMLM, EMMAX, GEMMA, and CMLM under the situations of five subpopulations and polygenic background control (kinship matrix) (Supplementary Data Set 3). As more than 10,000 QTNs were identified by GEMMA for HSW in 2018, the relevant results were not used in the subsequent analysis. As a result, 208 significant QTNs were identified for the above traits. Thirteen significant QTNs were simultaneously identified in two environments by two GWAS methods (Supplementary Table 3; Supplementary Data Set 4), some significant QTNs are presented in Figure 2. For example, Chr10-25206533-25223155 (LOD = 15.40∼37.89, *P* = 3.16E-08∼5.15E-09) was detected in 2018 and 2020 by MLM, EMMAX, and 3VmrMLM to be associated with HSW, SW, and SL (Table 1; Figures 2A–F), and the Q-Q plot in the Supplementary Figures 1A–D, which was corresponding to the GWAS results in Figure 2, except 3VmrMLM. And Chr1-71543546 (LOD = 7.70∼12.44) was detected in 2018 and 2020 by 3VmrMLM to be associated with SW (Supplementary Table 3). These QTNs were distributed on chromosomes 1–4, and 10 (≥20 QTNs for each chromosome) and had a 1.15% average proportion of their total phenotypic variation explained by each QTN, and there were 47, 115, and 46 QTNs, respectively, for HSW, SL, and SW (Supplementary Data Set 4).

**TABLE 1:** Eight key candidate genes derived from genome-wide association studies for seed-related traits.

Trait	Genome-wide association studies					Comparative genomics				Function	Reference
				
	Chromosome	Position (bp)	LOD score or *P*_1_-value	*r*^2^ (%)	Method	Candidate genes	*P*_2_-value	log_2_FC	Arabidopsis homologs		
**Single_env: Detection of main-effect QTNs for seed size-related traits**											
2018-HSW	1	52015258	21.84	0.81	3VmrMLM	EVM0016442/IAR1	0.05*	0.39	AT1G68100	IAA-alanine resistance protein 1	Rampey et al., 2013
	4	36876485	35.25	1.3	3VmrMLM	EVM0019602/flo2	0.02*	1.09	AT4G36920	Seed development	She et al., 2010
	11	3018112	25.95	2.62	3VmrMLM	EVM0010067/ABA2	0.18	0.21	AT1G52340	Seed maturation	Chauffour et al., 2019
2020-HSW	1	8177726	28.09	1.31	3VmrMLM	EVM0032114/KIX8	0.03*	0.49	AT3G24150	Seed development	Li et al., 2019
	4	7755858	19.6	1.03	3VmrMLM	EVM0015332/SUC4	0.02*	0.29	AT1G09960	Sucrose transport protein SUC4	Xu and Liesche, 2021
	10	25206533	15.41	0.59	3VmrMLM	EVM0015812/Emp24	0.02*	0.67	AT1G26690	Emp24 family protein	Ren et al., 2019
2018-SW	1	71543546	12.44	1.65	3VmrMLM	EVM0002784/BEE3	0.01*	1.24	AT1G73830	Seed development	Moreno et al., 2018
2020-SW	1	30724948	29.81	1.74	3VmrMLM	EVM0033315/SHB1	0.15	0.04	AT4G25350	Seed development	Zhang H. et al., 2017
	1	71543546	7.70	0.57	3VmrMLM	EVM0002784/BEE3	0.01*	1.24	AT1G73830	Seed development	Moreno et al., 2018
	6	13463604	12.93	0.55	3VmrMLM	EVM0028931/ZIP6	0.02*	−0.85	AT2G30080	Seed development	Lee et al., 2021
	9	24007163	61.96	5.8	3VmrMLM	EVM0027211/PAT14	0.03*	1.19	AT3G60800	Leaf senescence	Zhao et al., 2016
2018-SL	3	34837582	3.24E-08	NA	EMMAX	EVM0028440/ABI5	0.19	0.25	AT2G36270	ABSCISIC ACID-INSENSITIVE 5 isoform X4	Lynch et al., 2022
	6	1650897	1.92E-08	NA	EMMAX	EVM0030447/IKU2	0.43	0.78	AT3G19700	Embryo development	Xiao et al., 2016
	10	25223155	5.15E-09	0.992	CMLM	EVM0015812/Emp24	0.01*	0.67	AT1G26690	Emp24 family protein	Ren et al., 2019
	10	25222572	1.91E-06	0.515	CMLM	EVM0015812/Emp24	0.01*	0.67	AT1G26690	Emp24 family protein	Ren et al., 2019
	10	25223133	9.34E-09	2.264	CMLM	EVM0015812/Emp24	0.01*	0.67	AT1G26690	Emp24 family protein	Ren et al., 2019
	10	25223155	3.16E-08	3.411	CMLM	EVM0015812/Emp24	0.01*	0.67	AT1G26690	Emp24 family protein	Ren et al., 2019
	10	25223133	9.34E-09	NA	EMMAX	EVM0015812/Emp24	0.01*	0.67	AT1G26690	Emp24 family protein	Ren et al., 2019
**Multi_env: Detection of main-effect QTNs for seed size-related traits**											
HSW	1	8161305	36.33	0.8	3VmrMLM	EVM0032114/KIX8	0.03*	0.50	AT3G24150	Seed development	Li et al., 2019
	1	52015258	13.52	0.12	3VmrMLM	EVM0016442/IAR1	0.06	0.39	AT1G68100	IAA-alanine resistance protein 1	Rampey et al., 2013
	4	7755858	28.43	0.66	3VmrMLM	EVM0015332/SUC4	0.02*	0.30	AT1G09960	Sucrose transport protein SUC4	Xu and Liesche, 2021
	4	36876485	71.71	0.95	3VmrMLM	EVM0019602/flo2	0.02*	1.09	AT4G36920	Seed development	She et al., 2010
	10	25222572	37.89	0.67	3VmrMLM	EVM0015812/Emp24	0.01*	0.67	AT1G26690	Emp24 family protein	Ren et al., 2019
SL	1	8347626	24.09	0.35	3VmrMLM	EVM0032114/KIX8	0.03*	0.50	AT3G24150	Seed development	Li et al., 2019
	4	19559337	16.8	0.32	3VmrMLM	EVM0022984/flo2	NA	NA	Os04g0645100	Seed development	She et al., 2010
	10	25223133	29.75	0.64	3VmrMLM	EVM0015812/Emp24	0.01*	0.67	AT1G26690	Emp24 family protein	Ren et al., 2019
SW	6	13463604	27.54	1.62	3VmrMLM	EVM0028931/ZIP6	0.02*	−0.85	AT2G30080	Seed development	Lee et al., 2021

The *P*_1_-values were calculated by CMLM, EMMA, and 3VmrMLM, The *P*_2_-values were calculated using paired t-test from the average FPKM values at three stages between two high seed weight (*n*_1_ = 2) and tow seed weight (*n*_2_ = 2) mungbeans, and their significances were marked by * (0.05 level); FC and NA represent fold change and no expression, respectively.

#### Detection of quantitative trait nucleotides for seed-size-related traits in multiple environments

To detect more stable QTNs, three seed-size-related traits of 196 mungbean accessions measured in 2018 and 2020 were used to associate with 3607508 SNP markers using two-environment 3VmrMLM joint analysis. As a result, 32, 33, and 18 significant QTNs were identified for HSW, SL, and SW, respectively (Supplementary Table 3), and had a 1.08% average proportion of total phenotypic variation explained by each QTN. Moreover, eight significant QTNs were identified (Supplementary Table 4). For example, Chr1-8161305-8347626 (LOD = 24.09∼36.33) and Chr10-25222572-25223133 loci (LOD = 29.75∼37.89) were detected to be associated with HSW and SL, respectively (Supplementary Tables 3, 4).

Based on all the above main-effect QTNs in single- and multiple-environment analysis, five stable QTNs across various methods and/or two environments were found (Supplementary Table 5), including Chr1-8161305-8347626 (LOD = 24.09∼36.33), Chr2-12602704 (LOD = 17.71∼38.08), Chr4-10069367 (LOD = 17.72∼34.19), Chr5-10834954 (LOD = 9.53∼30.03), and Chr10-Chr10-25222572-25223133 (LOD = 29.75∼37.89), especially, Chr1-8161305-8347626 and Chr10-25222572-25223133 were simultaneously identified across methods and two environments.

#### Detection of quantitative trait nucleotide-by-environment interactions for seed-size-related traits in multiple environments

All the above datasets in GWAS were used to detect QEIs using 3VmrMLM. As a result, 5, 10, and 5 significant QEIs were found to be associated with HSW, SL, and SW, respectively (Supplementary Figure 2; Table 2). Among these QEIs, 5 had zero dominant-by-environment interaction effects, and 7 had zero additive-by-environment interaction effects. For example, the two loci Chr4-26262890 and Chr4-31677341 for HSW had only additive-by-environment interaction effects of 0.12 (Supplementary Figures 2A–C, LOD = 12.70; *r*^2^ = 0.26) and 0.08 (Supplementary Figures 2A–C, LOD = 12.65; *r*^2^ = 0.27), respectively.

**TABLE 2:** Twenty significant QTN-by-environment interactions for seed-size-related traits under multi-environments.

Trait	3VmrMLM						Candidate genes		*P*-value	log_2_FC	Arabidopsis homologs	Function	References
							
	Chr	Position (bp)	LOD (QE)	Add × Env1	Dom × Env1	*r*^2^ (%)							
HSW	1	25048694	7.99	0.08		0.18	EVM0010707; EVM0020394	EVM0010707	0.11	0.05	NA	NA	
	3	5498494	14.34	0.11		0.33	EVM0013436; EVM0027482; EVM002290	EVM0013436	0.21	1.53	AT3G61060	F-box protein PP2-A13	
	4	30176682	15.23	0.12		0.38	EVM0013210	EVM0013210/ FATB	0.09	0.50	AT1G08510	FATB	Bonaventure et al., 2003; Sun et al., 2014
	4	42563100	6.50	0.08		0.15	EVM0019039; EVM0011516	EVM0019039/ GSO1	0.09	0.91	AT4G20140	Seed development	Creff et al., 2019
	5	8962133	10.49	0.09		0.23	EVM0027740; EVM0007126	EVM0007126	0.05	−4.53	AT1G21450	Seed development	
SL	1	155976	12.73	0.00	−0.61	0.25	EVM0006618; EVM0002787; EVM0025368; EVM0002245; EVM0007007	EVM0006618	0.00	0.43	AT3G59910	Ankyrin repeat protein SKIP35 isoform X1	
	1	35982911	13.25	0.00	0.44	0.27	EVM0014255	EVM0014255	NA	NA	AT3G26570	Inorganic phosphate transporter 2-1, chloroplastic	
	4	22723706	12.93	−0.01	−0.61	0.26	EVM0015688	EVM0015688	0.03	0.07	AT5G50920	Chaperone protein ClpC, chloroplastic	
	4	26262890	12.70	0.00	−0.43	0.26	EVM0003123; EVM0001918	EVM0003123	NA	NA	NA	Citrate-binding protein-like	
	4	31677341	12.65	0.00	−0.61	0.27	EVM0009176; EVM0033509; EVM0023714; EVM0033630; EVM0032994	EVM0033630	0.03	NA	AT3G57520	Probable galactinol–sucrose galactosyltransferase 2 isoform X2	
	4	40101763	13.31	−0.01	−0.61	0.29	EVM0000524; EVM0025504	EVM0000524	0.21	NA	AT4G33140	Uncharacterized protein	
	7	16074671	12.90	0.01	−0.61	0.25	EVM0007632; EVM0003451; EVM0005587; EVM0017922; EVM0009325	EVM0007632	0.14	0.66	AT5G10330	Histidinol-phosphate aminotransferase, chloroplastic	
	7	28608053	12.99	−0.01	−0.61	0.27	EVM0025691; EVM0014665	EVM0025691	NA	NA	AT2G34930	Hypothetical protein	
	8	32848165	12.70	0.00	−0.61	0.26	EVM0033747; EVM0012210; EVM0020228; EVM0006042; EVM0026839; EVM0012261; EVM0001209; EVM0016212; EVM0027531; EVM0030105; EVM0021224; EVM0011572	EVM0012210/ LACS2	0.03	−2.53	AT1G49430	Long chain acyl-CoA synthetase 2 isoform X1	Schnurr et al., 2004; Bai et al., 2022
	11	24829262	12.65	0.00	−0.61	0.25	EVM0006035; EVM0003000; EVM0020076; EVM0004982	EVM0020076	0.03	0.22	AT1G59870	ABC transporter G family member 36	
SW	2	29996834	9.66	0.02	0.26	0.62	EVM0004520; EVM0005114	EVM0004520	0.09	1.02	AT3G09300	Oxysterol-binding Protein-related protein 3B	
	4	5255551	7.38	0.02	−0.12	0.48	EVM0010724; EVM0028229	EVM0010724	0.11	NA	AT1G80550	Pentatricopeptide repeat-containing protein	
	4	19640302	16.41	0.00	−0.39	1.17	NA	NA	NA	NA	NA		
	7	18410421	9.28	−0.03	−0.20	0.61	EVM0022194; EVM0018119; EVM0020361; EVM0025547	EVM0022194	0.08	0.47	AT1G68690	Proline-rich receptor-like protein kinase PERK9	
	9	24007163	61.96	−0.14	−0.10	5.80	EVM0027211; EVM0026090; EVM0028888; EVM0024624; EVM0026781; EVM0029904; EVM0012085; EVM0004220	EVM0027211/ PAT14	0.03	1.19	AT3G60800	Leaf senescence	Zhao et al., 2016

The *P*-values were calculated using paired *t*-test from the average RPKM values at three stages between two high seed weight (*n*_1_ = 2) and tow seed weight (*n*_2_ = 2) mungbeans, and their significances were marked by * (0.05 level); FC and NA represent fold change and no expression, respectively.

The two loci Chr1-155976 and Chr1-3598291 for HSW had only dominant-by-environment interaction effects of −0.61 (LOD = 12.73; *r*^2^ = 0.25) and 0.44 (LOD = 13.25; *r*^2^ = 0.27), respectively. Among the 20 QEIs, the loci Chr4-5255551 and Chr7-16074671 had inconsistent directions between additive- and dominant-by-environment interaction effects.

In addition, among these QEIs, the QEI locus Chr9-24007163 for SW had large effect, and *r*^2^ was 5.8% (Supplementary Figure 2B, LOD = 61.95). The additive and dominant effects in environment 1 were −0.14 and −0.098, respectively.

#### Candidate genes for seed-size-related traits

**FIGURE 3:**
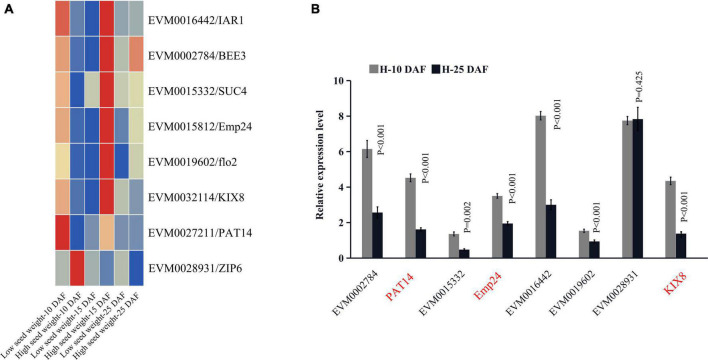
The expression of eight key candidate genes. The expression profiling of eight key candidate genes significantly associated with seed-size-related traits. The expression profiling of eight key candidate genes between two high-seed-weight and two low-seed-weight mungbeans **(A)**. Real-time PCR analysis of the eight key candidate genes; the *t*-test was used to test the significant differences of genes expression between two high-seed-weight mungbeans at 10 DAF and 25 DAF **(B)**. DAF, days after flowering.

A total of 6912 DEGs were identified between two high-seed-weight and low-seed-weight mungbeans (FDR ≤ 0.05) (Supplementary Figures 3A,B; Supplementary Data Set 6). These DEGs were intersected with 809 genes around significant QTNs for HSW, SL, and SW (Supplementary Tables 3, 4; Supplementary Data Sets 4, 5). As a result, 53 out of 809 genes were differentially expressed (*P* ≤ 0.05, Log_2_FC ≥ 0.5). Using comparative genomics analysis, 12 out of 53 DEGs were homologous to previously reported seed development related genes in rice and *Arabidopsis thaliana*, in which *KIX8*, *PAT14*, *Emp24/25*, *IAR1*, *BEE3*, *SUC4*, *flo2*, and *Zip6* had been confirmed *via* functional analysis in rice and *A. thaliana* (Table 1), such as *VrKIX8* (LOD = 24.09∼36.33), *VrEmp24/25* (LOD = 15.40∼37.89, *P* = 3.16E-08∼5.15E-09), *VrPAT14* (LOD = 61.96), and *VrZIP6* (LOD = 27.54). Among the eight genes, *VrKIX8*, *VrEmp24/25*, *VrIAR1*, *VrBEE3*, *VrSUC4*, and *Vrflo2* were significantly upregulated in high-HSW accessions, *VrPAT14* was significantly downregulated, and *VrZIP6* had no significant difference (Figure 3A), as compared to those in low-HSW accessions using the transcriptome data at 10, 15, and 25 DAF (Supplementary Data Set 4). We conducted RT-qPCR analysis to further confirm the eight key candidate genes. The results showed that seven genes were confirmed, except *VrZIP6*, a transcription factor related to seed development. All the seven genes had higher expression levels in the early stage of seed development (10 DAF) than in the late maturation stage of seed development (25 DAF) (Figure 3B; Supplementary Data Set 7), indicating their essential roles at early stage of seed development.

Using the same approach described above, among 65 genes around 20 QEIs, four were homologous to previously reported seed development related genes in rice and *A. thaliana* (Table 2), although new experiments are necessary to explore these novel GEI-trait associations. The four genes were described as below. *VrFATB* was linked to the locus Chr4-30176682 (Supplementary Figure 2A). As described in Bonaventure et al. (2003) and Sun et al. (2014), *FATB* is a major determinant of saturated fatty-acid synthesis, and increases *FATB* activity at low temperature during seedling establishment caused high saturated fatty-acid content in plant. *VrGSO1* was linked to the locus Chr4-42563100 (Supplementary Figure 2A). As observed in Creff et al. (2019), *GSO1* was a stress signal-pathway-related gene, and stress-associated *MPK6* protein acted downstream of *GSO1* in developing embryo. *VrPAT14* was linked to the locus Chr9-24007163 (Supplementary Figure 2B). In Zhao et al. (2016), *PAT14* was involved with NPR1-dependent salicylic-acid signaling. *VrLACS2* was linked to the locus Chr8-32848165 (Supplementary Figure 2C), in which *VrLACS2* was essential for normal cuticle development in *Arabidopsis* (Schnurr et al., 2004) and *CrLACS2* suppression resulted in 50% less oil, yet with a higher amount of chloroplast lipids under N-deprivation (Bai et al., 2022).

#### Haplotype analysis of the main candidate genes

**FIGURE 4:**
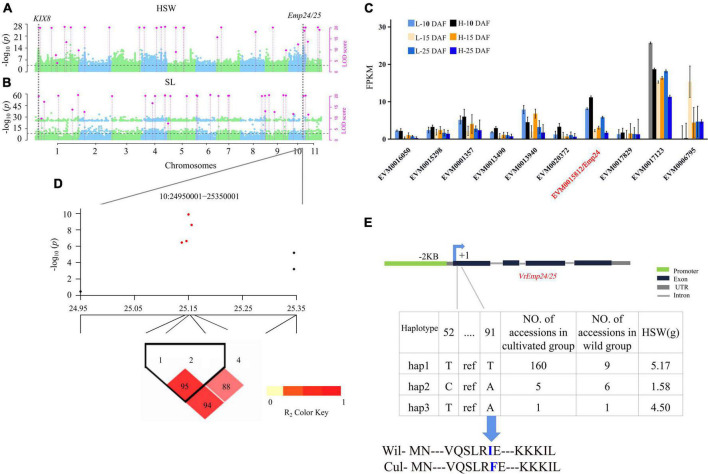
Genetic analysis of *VrEmp24/25*. Local Manhattan plots for HSW under multi-environments. LOD ≥ 3.0 for the 3VmrMLM as the significant QTN **(A,B)**. The expression profiling of 10 candidate genes for HSW identified at 30 Kb around Chr10-25222572-25223133 loci in the seed between two high-seed-weight and two low-seed-weight mungbeans **(C)**. LD heatmaps surrounding Chr10-25222572-25223133 loci **(D)**. Haplotype analysis of *VrEmp24/25* **(E)**, the thirtieth amino acid of *VrEmp24/25* changed from ATT (Ile, I) to TTT (Phe, F). DAF, days after flowering. Wil, the wild accessions. Cul, the cultivated accessions.

Two DEGs, *VrEmp24/25* and *VrKIX8*, were detected in the single- and multi-environment analyses (Figures 4A,B), and verified by RT-qPCR. Their haplotypic analyses were described as below.

In the haplotype analysis of *VrEmp24/25*, five SNP markers were found to be within *VrEmp24/25* and the promoter region (Supplementary Data Set 8), and the two SNP markers in *VrEmp24/25* were used to consist of three haplotypes (Figure 4D). Among the three haplotypes, hap 1 (5.17 g) had significantly higher HSW than hap 2 (1.58 g) and hap 3 (4.50 g; *P* = 2.11E-29) (Supplementary Table 7). Thus, hap 1 is elite haplotype. And the elite haplotypes TT made up more than 90.9% (160/176) in the cultivated mungbeans. *VrEmp24/25* with elite haplotype frequencies less than 45% in wild mungbeans (Supplementary Table 7; Figure 4) can be exploited for the improvement of mungbean cultivars.

**FIGURE 5:**
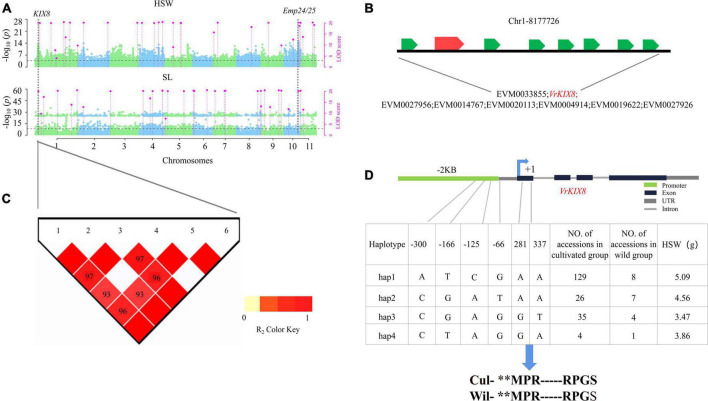
Genetic analysis of *VrKIX8*. Local Manhattan plots for HSW in multi-environments. LOD ≥ 3.0 for the 3VmrMLM as the significant QTN **(A)**. LD heatmaps surrounding Chr1-8161305-8347626 loci **(B)**. Genes around the significant QTN region, shown at the bottom **(C)**. Haplotype analysis of *VrKIX8* **(D)**. Wil, the wild accessions. Cul, the cultivated accessions. symbol “**” means omit the same sequence part.

Around the significant QTN Chr1-8161305-8347626 (Figure 5A; Supplementary Data Set 8), eight genes were found distributed in the region (Figure 5B). And six polymorphic loci, i.e., Chr1_8243935, Chr1_8243938, Chr1_8243939, Chr1_8243940, Chr1_8243945, and Chr1_8244001 were found in *VrKIX8* and the promoter region. All the six SNP were used to conduct the haplotype analysis (Figure 5C). Among the three haplotypes, hap 1 (5.09 g) had significantly higher HSW than hap 2 (4.56 g), hap 3 (3.47 g), and hap 4 (3.86 g) (Supplementary Table 7). Thus, hap 1 is elite haplotype. The elite haplotypes ATCGAA made up more than 73.2% (129/176) in the cultivated mungbeans, while the haplotype frequencies of CGAGT and CTAGGA were more than 25% (5/20) in wild mungbeans. Though Chr1_8243945 and Chr1_8244001 were located within the 5′ UTR of *VrKIX8*, and the amino acid sequence had not changed between cultivated mungbeans and wild mungbeans (Figure 5D). The SNP in 5′ UTRs could influence the translation efficiency of *VrKIX8* (Evfratov et al., 2017). The HSW in hap 1 (5.16 g) was significantly higher than that in hap 2 to hap 4 (3.50–4.66 g; *P* = 1.19E-21).

Based on these results, we deduced that these two SNP and six SNP cause the difference expression of the *VrEmp24/25* and *VrKIX8* gene, respectively. The discovery of *VrEmp24/25* and *VrKIX8* two domestication/improvement genes can accelerate breeding selections and facilitate ideal crop designs.

#### Expression patterns of seed development pathway genes in mungbean

**FIGURE 6:**
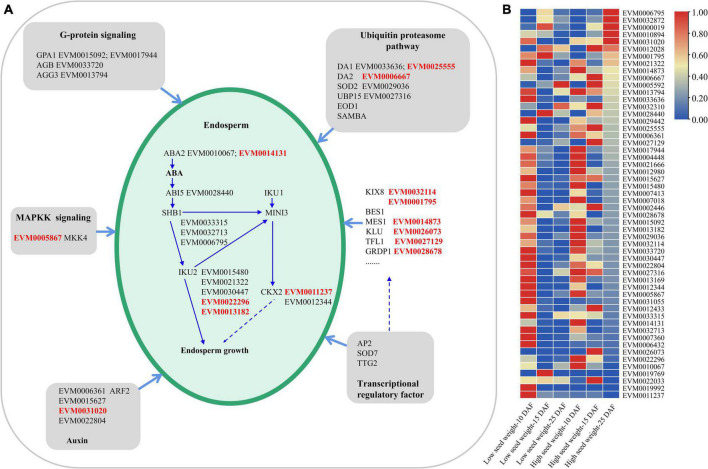
The seed development pathway in mungbean **(A)** and the expression profiling of 54 candidate genes predicted by comparative genomics, identified in this study **(B)**. GPA1, G PROTEIN ALPHA SUBUNIT 1; AGB, heterotrimeric G-protein beta subunit; AGG3, heterotrimeric G-protein gamma-subunit; DA1/DA2, a ubiquitin-activated peptides; SOD2, chloroplastic copper/zinc superoxide dismutase CSD2; EOD1, E3 ubiquitin ligase; UBP15, ubiquitin-specific protease; SAMBA, plant-specific negative regulator of the APC/C complex; MKK4, mitogen-activated map kinase; ABA2, ABA DEFICIENT 2; ABI5, ABA INSENSITIVE 5; SHB1, a nuclear and cytosolic protein; IKU1/IKU2, leucine rich repeat (LRR) kinase; CKX2, CYTOKININ OXIDASE 2; MINI3, MINISEED 3; ARF2, auxin response factor; AP2, AP2/EREBP (ethylene-responsive element-binding protein) class of transcription factors (Figure 5A). We also identified five transcription factors, three BES1 and two SOD7, transcription repressor involved in regulation of inflorescence architecture; KIX8, KINASE-INDUCIBLE DOMAIN INTERACTING8; MES1, METHYL ESTERASE 1; KLU, cytochrome P450 CYP78A5 monooxygenase. DAF, days after flowering; ABA, abscisic acid; DAF, days after flowering.

As seed development pathway genes were largely unknown in mungbean, we mined seed development pathway genes by comparative genomics and transcriptomics analysis. As a result, 54 genes in seed-development pathway were identified in this study (Figure 6; Supplementary Data Set 9), such as two *GPA1*, one *AGB*, and one *AGG3*. In the ubiquitin proteasome pathways, two *DA1*, one *DA2*, one *SOD2*, one *EOD1*, and one *UBP15* rather than *SAMBA* were identified. In the auxin pathways, two *ABA2*, one *ABI5*, three *SHB1*, five *IKU2*, and three *CKX2* rather than IKU1 and MINI3 were identified (Figure 6A). Five transcription factors including three *BES*1, and two *SOD7* were identified. Moreover, 16 genes for seed size developments were found to be with uncertain pathways, including three *KIX8*, five *MES1*, and one *KLU* (Figure 6A; Supplementary Data Set 9). Among the 54 genes, 13 genes were significantly differentially expressed (*P*-value < 0.05, *t*-test) between two low-seed-weight (nos. G169 and G171) and two high-seed-weight (no. G141 and G143) accessions in the 196 mungbean accessions using the transcriptome data at 10, 15, and 25 DAF (Figure 6B; Supplementary Data Set 8). Moreover, almost 90% of the 54 genes (48/54) had higher expressions in the early stage of seed development (10 and 15 DAF) than in the late maturation stage (25 DAF), including *VrKIX8* (EVM0032114), which was commonly identified in the GWAS by 3VmrMLM for HSW and SL. And EVM0010067/*VrABA2*, EVM0033315/*VrSHB1*, EVM0028440/*VrABI5*, and EVM0030447/*VrIKU2* were also identified in the GWAS by 3VmrMLM, within 100 Kb region of significant QTNs (Table 1).

We also did the PPI analysis among the seed development pathway genes, and found five pairs of PPIs were larger than the medium confidence value of 0.40 (Supplementary Table 7), indicating the existence of significant PPIs, i.e., EVM0013794.1 (VrAGG3) and EVM0006667.1 (VrDA2) (0.478), EVM0033720.1 (VrAGB) and EV944.1 (VrGPA1-1) (0.995), as well as EVM0033720.1 (VrAGB) and EVM0015092.1 (VrGPA1-2) (0.995).

## Discussion

The high-yield and efficiency breeding progress of mungbeans have been limited by the lack of ideal yield-related genes. At present, few QTNs or QTLs of yield-related traits in mungbeans have been reported (Kang et al., 2014). This study provided a genetic analysis of seed-size-related traits in mungbeans, to improve the accuracy of significant QTNs, we used multiple genome-wide M0017 association studies combined with multi-omics analysis to mine candidate genes associated with yield-related traits. Firstly, a total of 98 QTNs and 20 QEIs were identified using 3VmrMLM, while 95 and 15 QTNs were identified using EMMAX, and CMLM, respectively. Then, in the identification of candidate genes, 12 key candidate genes were mined, and seven of them including *VrKIX8*, *VrEmp24/25*, and *VrPAT14* were evidenced by transcriptome analysis and RT-qPCR analysis. Lastly, through haplotype analysis, the thirtieth amino acid of *VrEmp24/25* in the elite haplotype was changed from Ile to Phe. And there were six SNP in the promoter and 5′ UTRs of *VrKIX8*, however, the amino acid sequence of *VrKIX8* in the elite haplotype was not changed. The results provided the theoretical basis for both the functional identification of seed-size-related genes and for quality improvements in mungbean breeding.

### Multiple genome-wide association studies methods combined with multi-omics analysis in mining candidate genes

In the GWAS, how to identify candidate genes around significant QTNs has been a challenge. Liu et al. (2020c), Zhang et al. (2021), and Gong et al. (2022) selected the 100-kb interval upstream and downstream of the significant QTN as the candidate interval in watermelon and soybeans. Usually, the interval has been chosen according to the LD decay values.

In order to determine stable QTNs and key candidate genes for seed-size-related traits, we adopted the following analyses. Firstly, we used CMLM, EMMAX, GEMMA, and 3VmrMLM to identify stable QTNs, as a result, five stable QTNs for seed-size-related traits were detected in single- and multiple-environments (Supplementary Table 5), i.e., Chr1-8161305-8347626 (LOD = 24.09∼36.33), and Chr10-25222572-25223133 loci (LOD = 29.75∼37.89).

Second, in the identification of candidate genes, we conducted issue expression analysis, and comparative genomics analysis. 53 out of the 809 candidate genes were significantly differentially expressed between high and low HSW accessions (*P* ≤ 0.05, Log_2_FC ≥ 0.5). Among the 53 DEGs, *Arabidopsis* homologous genes of the 12 key candidate genes had certain molecular functions. Notably, 10 of those genes were identified by 3VmrMLM (Table 1). Seven key candidate genes (*VrKIX8*, *VrEmp24/25*, *VrIAR1*, *VrBEE3*, *VrSUC4*, *VrPAT14*, and *Vrflo2*) were significantly differentially expressed between the low-seed-weight and high-seed-weight accessions, and further verified by RT-qPCR analysis (Table 1; Figure 4). *VrKIX8* (Chr1-8161305-8347626) and *VrEmp24/25* (Chr10-25222572-25223133) may be main genes in controlling seed-size-related traits.

Notably, 3VmrMLM showed more powerful ability in the detection of significant QTN than GEMMA, EMMAX, and CMLM, as it found more differentially expressed key candidate genes than other methods. The combination of 3VmrMLM and multi-omics analysis in the genetic analysis of complex traits was helpful.

### Genome-wide association study provided potential genes *VrEmp24/25* and *VrKIX8* for mungbean seed-size-related traits

*VrEmp24/25* was an important seed-size traits related gene, the evidence was as below: Firstly, Chr10-25206533-25223155 locus for seed size traits was detected in 2018 and 2020 by CMLM, EMMAX, and 3VmrMLM (Figure 2), and there were 10 genes in its interval (Figure 4C). Secondly, among the 10 genes, only *VrEmp24/25* (EVM0015812) (*P* = 0.014, Log_2_FC = 0.67) had deferentially expressed across different phenotype accessions (Figure 4C; Supplementary Data Set 4). Besides, in maize, the loss function of *EMP24* and *Emp25* would impair embryo and endosperm development (Xiu et al., 2020). *EMP24* was required for the splicing of nad4 (Ren et al., 2019), and the lack of either Nad4 or Nad5 blocked the assembly of complex I holoenzyme in *Arabidopsis* (Ligas et al., 2019). The loss of the steady-state level of mitochondrial nad5 mature mRNA blocked the assembly of complex I and caused an arrest in endosperm development (Zhang Y. F. et al., 2017). Lastly, the elite haplotypes of *VrEmp24/25* (TT) made up the main proportion of more than 90.9% in cultivated mungbeans, 45% in wild mungbeans (Figure 4E). The HSW in hap 1 haplotypes accessions was significantly higher than that in hap 2 and hap 3 (*P* = 2.11E-29). It was reported that a single amino acid completely prevented the appearance of the enzyme in the medium, and we inferred that the related variation could lead to the change in enzyme activity (East et al., 1990; Alfson et al., 2018).

There have four evidences to take *VrKIX8* as another important seed-size trait gene. Firstly, *VrKIX8* associated with Chr1-8161305-8347626 (LOD = 24.09∼36.33) for HSW and SL were detected in multi-environment by 3VmrMLM (Figure 5A; Supplementary Table 5). Secondly, *VrKIX8* (LOD = 24.09∼36.33) had significantly differentially expressed between high- and low-HSW accessions (Figure 3A). Then, in *Arabidopsis*, the disruption of *KIX8/9* and *PPD1/2* could cause large seeds due to increased cell proliferation and cell elongation in the integuments (Liu et al., 2020a). In soybeans, the loss of the function *GmKIX8-1* showed a significant increase in the size of seeds and leaves. In addition, the increase in organ size was due to the increased cell proliferation, rather than cell expansion. *GmKIX8-1* showed negatively regulated cell proliferation in plants (Nguyen et al., 2021). Lastly, the elite haplotypes of *VrKIX8* (ATCGAA) made up the main proportion of more than 73% in cultivated mungbeans, 40% in wild mungbeans. Moreover, there are four SNPs in the promoter and of *VrKIX8*, and two SNPs in the CDS region, however the amino acid sequence did not change between the elite haplotypes and the other haplotypes (Figure 5C). The HSW in hap 1 haplotypes accessions was higher than that in hap 2 to hap 4 (*P* = 1.19E-21). We supposed that the mutations may have influenced the translation efficiency of *VrKIX8* and caused low expression in cultivated accessions during mungbean domestication.

### Genes participate in seed development progress

The genes controlling seed development progress in mungbean are largely unknown (Ha et al., 2021). In this study, we identified fifty-four candidate genes in the seed-development pathways, i.e., *aba2* (Cheng et al., 2014; Chauffour et al., 2019), ABI5 (Lynch et al., 2022), *SHB1*, MINI3, and IKU2 (Garcia et al., 2003; Xiao et al., 2016; Zhang H. et al., 2017), mutants of those genes induced abnormal seed development in *Arabidopsis*. And, five genes were also commonly identified *via* GWAS (Table 1). Those five genes (*VrKIX8*, *VrABA2*, *VrSHB1*, *VrABI5*, and *VrIKU2*) are more likely to be reliable, especially for *VrKIX8*, as described above.

We also analyze the possible correlation between the main seed development pathways. Among the 54 genes, five genes (*VrAGG*, *VrDA2*, *VrAGB*, *VrGPA1-1*, and *VrGPA1-2*) consisted of five pairs of significant PPIs. Interestingly, four pairs PPIs were found to be in the G-protein-signaling pathway, and one pair of PPIs was found to be in the G-protein-signaling and the ubiquitin proteasome pathways (Figure 6; Supplementary Table 6). Ubiquitin proteasome pathway is an important pathway for the selective degradation of proteins and seed development (Smalle and Vierstra, 2004), and the G-protein-signaling pathway is a ubiquitous cell transmembrane signal transduction pathway in eukaryotes (Huang et al., 2006). Moreover, mutations in *GPA1* or *AGB1* could cause short flowers (Lease et al., 2001; Ullah et al., 2001). The overexpression of *AGG3* promoted seed and organ growth by increasing cell proliferation, and loss-of-function mutations in *AGG3* caused small seeds and organs (Chakravorty et al., 2011; Li et al., 2012). The ubiquitin receptor DA1 could control seed size by restricting cell proliferation in maternal integuments (Li et al., 2008). DA1 functioned synergistically with DA2 to restrict seed growth, and DA2 physically interacted with DA1 *in vitro* and *in vivo* (Song et al., 2007; Xia et al., 2013). This interaction could mediate the interactions between the G-protein-signaling pathway and the ubiquitin proteasome pathway, which might offer an important clue in the mechanism analysis of seed development.

In addition, 48 genes had higher expressions in the early stage of seed development than in the late maturation stage of seed development, indicating that seed-development-related genes function primarily in the early stages of seed development, which was consistent with the findings of Zuo et al. (2022) in soybean.

## Conclusion

This study conducted GWAS for seed-size-related traits in mungbeans. 98 QTNs and 20 QEIs were identified using 3VmrMLM, while 95, >10,000, and 15 QTNs were identified using EMMAX, GEMMA, and CMLM, respectively. A total of 12 key candidate genes were mined, which were homologous to known seed-development genes in rice and *A. thaliana*. *VrEmp24/25* and *VrKIX8* were identified as main candidate genes around two stable QTNs, the two candidate genes were further confirmed by RT-qPCR and haplotype analysis, and prevalent haplotypes of *VrEmp24/25* and *VrKIX8* may be useful in mungbean breeding.

## Data availability statement

The datasets presented in this study can be found in online repositories. The names of the repository/repositories and accession number(s) can be found below: The WGS sequencing data of 196 mungbean accessions was uploaded to NGDC, with subCRA011538, subSAM100395, and PRJCA010704 ID.

## Author contributions

JL, XY, and XC conceived of the project and its components. JL, JC, and YL performed the field experiments. JL, QY, CX, and RW performed the bioinformatics analysis and real data analysis. JL, XC, and XY wrote and revised the manuscript. All authors reviewed the manuscript.

## Footnotes

1
http://bio-bwa.sourceforge.net/bwa.shtml

2
https://gitee.com/mirrors/GATK

3
http://dalexander.github.io/admixture/download.html

4
http://zzlab.net/GAPIT

5
http://csg.sph.umich.edu//kang/emmax/download/index.html

6
https://github.com/genetics-statistics/GEMMA

7
https://github.com/YuanmingZhang65/IIIVmrMLM

8
http://www.usadellab.org/cms/index.php?page=trimmomatic

9
https://string-db.org//

## Abbreviations

GWAS: genome-wide association study
HSW: 100-seed weight
FPKM: Fragments Reads Per Kilobases per Million reads
PPI: protein–protein interaction
RNA-seq: RNA sequencing
QEIs: QTN-by-environment interactions
GEMMA: genome-wide efficient mixed-model association
CMLMs: compressed mixed linear models
EMMAX: efficient mixed-model association expedited
KIX8: KINASE-INDUCIBLE DOMAIN INTERACTING8
Emp24/25: emp24/gp25L/p24 family
QTNs: quantitative trait nucleotides
SNP: single nucleotide polymorphism
SW: seed width
SL: seed length.
