# Supplementary material for: Genome-wide association studies provide genetic insights into natural variation of seed-size-related traits in mungbean
Source: Front Plant Sci. 2022 Oct 13;13:997988. doi: 10.3389/fpls.2022.997988 (PMC9608654; doi:10.3389/fpls.2022.997988)
Supplement: Supplementary file 1 [file Table_1.pdf]

SUPPORTINGINFORMATION

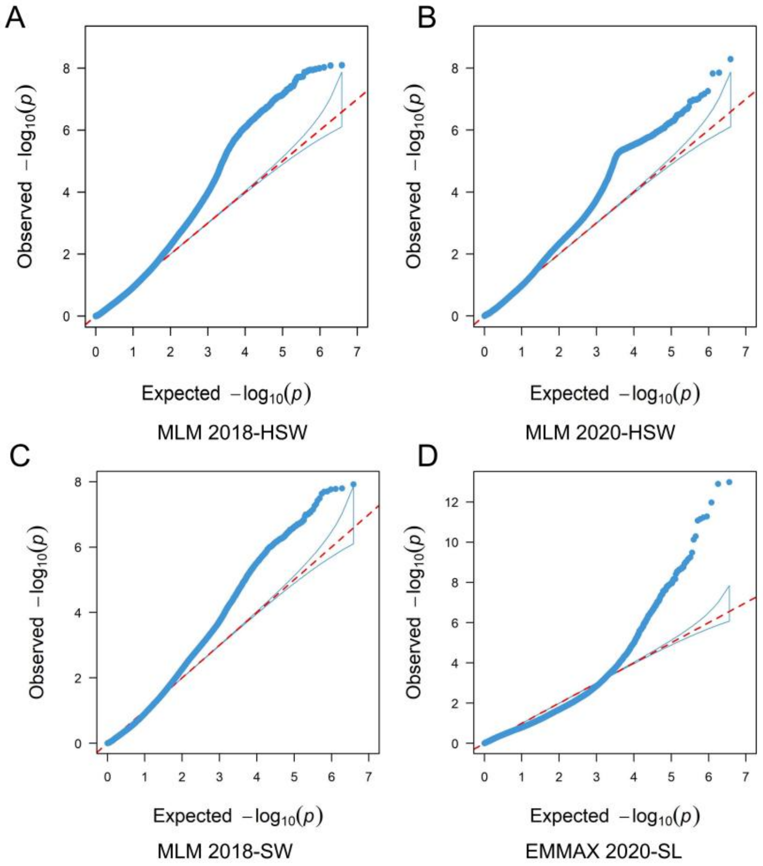

Supplemental Figure 1. Quantile-quantile plots for the GWAS of seed-size-related traits for HSW (A-B), SW (C) and SL (D), which was corresponding to the part of GWAS results in Figure 2. The red line indicates the signifcance threshold (−log10(P)=6.56) (color fgure online).

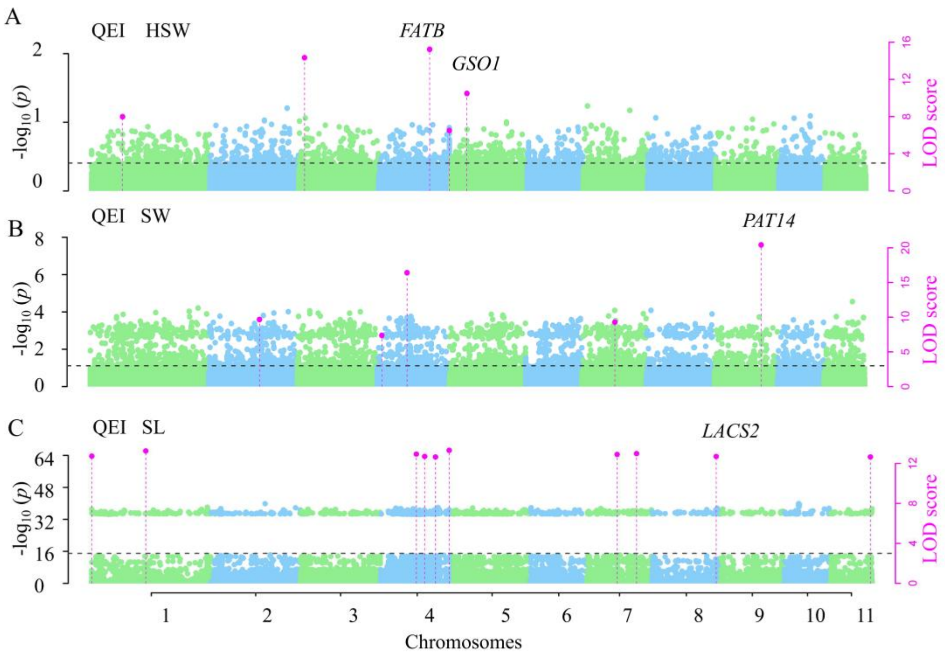

Supplementary Figure 2. The significant and suggested QEIs for seed-size-related traits in multiple environments. GWAS for HSW (a), SW (b) and SL (c). For significant QEIs with LOD ≥ 3.0, the critical values are marked by horizontal lines. The Y-axis on the left side reports -log10 P-values of QEIs while Y-axis on the right side reports LOD scores, which were obtained from likelihood ratio test for significant QEIs. These LOD scores are shown in points with straight lines.

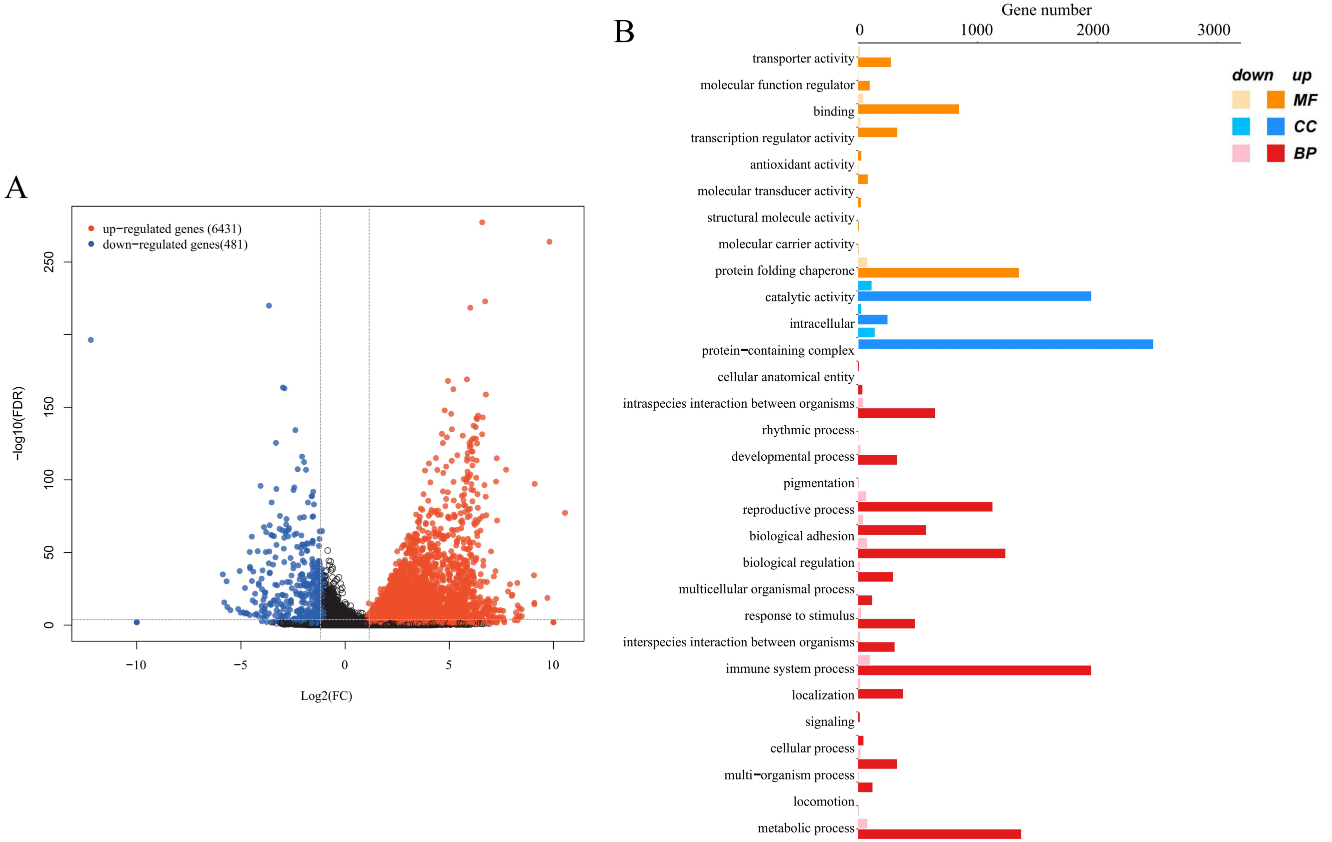

Supplementary Figure 3. A total of 6912 DEGs identified between two big-seed and two small-seed mungbeans. (A) Volcano plots are used for visualizing differential expression genes between the above two groups. The vertical lines correspond to 2.0-fold ($\log_2$ fold-change). Red and blue points represent the significant DEGs with FDR $\leq$ 0.05, and $\log_2$(fold-change) > 1. (B) Top 30 pathways of KEGG functional enrichment among DEGs. Coloring indicates P-value with higher in blue and lower in red. The lower P-value indicates more significantly enriched. Column length indicates DEG number.

Supplemental Table 1. The list of primers used in this study.

Primer	Sequence (5'to3')	Purpose	Products (bp)
EVM0015812 1F	GGTTTTTTGTTCTGATTTTTGGACT	Quantitative Real time RT-qPCR	139
EVM0015812 1R	TTAATGATACTGTAATTGCCCACTG	Quantitative Real time RT-qPCR	
EVM0016442 2F	TGGTCATTCACATTCTTTGGCCGAT	Quantitative Real time RT-qPCR	146
EVM0016442 2R	TCTTCAACATACCTCACTACCTTCT	Quantitative Real time RT-qPCR	
EVM0002784 3F	CTACAGATAGTCACAGTTTAGCAGA	Quantitative Real time RT-qPCR	151
EVM0002784 3R	GCACGTAGTTTATGATCTCGTCCAA	Quantitative Real time RT-qPCR	
EVM0027211 4F	GTTGGTTCTTGGGGTCGTTGGTGTG	Quantitative Real time RT-qPCR	94
EVM0027211 4R	AACAAGAGAATCAAGTCCTCCAGCA	Quantitative Real time RT-qPCR	
EVM0015332 5F	GTTTTCGTTTTCGGGTTTTGGATTT	Quantitative Real time RT-qPCR	141
EVM0015332 5R	AGCCATAAACAGGGAGAAATAAGCA	Quantitative Real time RT-qPCR	
EVM0019602 6F	GCAGAGGAGAGACAGATTCGGGATA	Quantitative Real time RT-qPCR	161
EVM0019602 6R	AATCTCCAACACTTTCTTCATACGA	Quantitative Real time RT-qPCR	
EVM0028931 7F	TTTCCATCTTCACAATCTTCGTAAC	Quantitative Real time RT-qPCR	138
EVM0028931 7R	CAAGAATAACACCCGCAGCAAAACA	Quantitative Real time RT-qPCR	
EVM0032114 8F	TCGCAACACTGAAGAGGTTCGCAAT	Quantitative Real time RT-qPCR	111
EVM0032114 8R	GCTTGATGTTCCACGCAATGTTTTT	Quantitative Real time RT-qPCR	
At3g18780-F	*TTCTTTATGGTTGGGTTTGC*	Quantitative Real time RT-qPCR	152
At3g18780-R	*GCTCGTCTACCTCCTTTGTG*	Quantitative Real time RT-qPCR	

F: forward primers; R: reverse primers. Primers were designed by NCBI, and tested by RCR of Tubulin.

Supplemental Table 2. Phenotypic characteristics for seed-size-related traits in 196 mungbean accessions.

Traits	Year	Mean	Std Dev	Minimum	Maximum	Skewness	Kurtosis	CV (%)
HSW (g)	2018	4.87	1.88	0.87	8.95	-0.17	-0.82	0.38
	2020	5.03	1.97	0.68	9.49	-0.14	-0.90	0.39
SW (mm)	2018	3.51	0.49	1.79	4.29	-0.92	0.81	0.14
	2020	3.48	0.52	1.69	4.25	-0.96	0.77	0.15
SL (mm)	2018	4.69	0.73	2.73	6.28	-0.28	-0.43	0.16
	2020	4.70	1.17	2.66	15.49	5.09	47.38	0.17

Supplemental Table 3. Eighty-three main-effect QTNs for seed size-related traits using multi-environment 3VmrMLM.

Trait	3VmrMLM				Candidate genes		P-value	log_2FC	Arabidopsis homologs	Functional Annotation
	Chromosome	Position (bp)	LOD	r^2(%)						
HSW	1	8161305	36.33	0.80	EVM0020113;EVM0033855;EVM0027956;EVM001	EVM0032114/KIX8	0.03	0.50	AT3G24150	seed development
	1	12166173	20.26	0.34	EVM0003555;EVM0017999;EVM0026562;EVM001	EVM0003555	0.01	1.18	NA	LOW QUALITY
	1	30702036	19.99	0.48	EVM0004245;EVM0030502;EVM0033315;EVM003	EVM0030502	0.24	0.22	NA	uncharacterized protein
	1	36639870	7.63	0.17	NA	NA	NA	NA	NA	NA
	1	39199671	4.04	0.05	EVM0015701	EVM0015701	NA	NA	NA	hypothetical protein
	1	49690456	64.12	0.61	EVM0025605	EVM0025605	0.09	0.69	AT4G39080	V-type proton ATPase
	1	52015258	13.52	0.12	EVM0024147;EVM0014950;EVM0016442;EVM001	EVM0016442/IAR1	0.06	0.39	AT1G68100	IAA-alanine resistance
	1	57388320	41.53	1.14	EVM0005997;EVM0022334;EVM0017333	EVM0005997	NA	NA	NA	hypothetical protein
	1	71130643	9.79	0.21	EVM0013852;EVM0011488;EVM0018379;EVM003	EVM0013852	0.26	-0.21	AT2G32230	proteinaceous RNase P 1,
	2	12602704	38.08	0.77	NA	NA	NA	NA	NA	NA
	2	47643044	36.14	0.14	EVM0007375;EVM0033365;EVM0008522;EVM000	EVM0007375	0.32	0.24	AT4G36470	theobromine synthase
	3	44112748	34.25	0.84	EVM0006439;EVM0030529;EVM0018588	EVM0006439	0.07	0.33	AT3G10330	transcription initiation
	4	7755858	28.43	0.66	EVM0028371;EVM0015332	EVM0015332/SUC4	0.02	0.30	AT1G09960	sucrose transport protein
	4	25112250	20.05	0.17	EVM0000194	EVM0000194	0.18	0.20	AT2G13690	BTB/POZ
	4	31168722	50.19	1.36	EVM0025782;EVM0025999;EVM0030918;EVM001	EVM0025782	0.07	0.34	AT2G36850	callose synthase 10 isoform
	4	36876485	71.71	0.95	EVM0019602	EVM0019602/flo2	0.02	1.09	AT4G36920	seed development
	5	10834954	30.03	0.24	EVM0025690;EVM0007856;EVM0019576	EVM0025690	NA	NA	NA	uncharacterized protein
	5	10880212	9.06	0.20	EVM0030862	EVM0030862	0.11	0.49	AT4G08180	oxysterol-binding
	5	19699349	25.06	0.51	EVM0017152;EVM0025307;EVM0033674;EVM000	EVM0017152	0.17	NA	AT5G38280	rust resistance kinase
	5	20335821	23.73	0.37	EVM0023970	EVM0023970	0.11	-3.31	NA	uncharacterized protein
	7	848566	15.72	0.22	EVM0028338;EVM0012440;EVM0000261;EVM000	EVM0028338	0.43	0.05	AT5G14580	polyribonucleotide
	7	7420121	38.73	0.66	EVM0012382;EVM0019250;EVM0026655;EVM001	EVM0012382	0.40	-0.25	AT5G49160	DNA
	8	18575610	18.17	0.16	EVM0006802	EVM0006802	NA	NA	NA	uncharacterized protein
	9	9193891	33.84	0.95	EVM0005358;EVM0014672;EVM0009150	EVM0005358	0.40	-0.20	NA	uncharacterized protein

	9	28467689	9.84	0.20	EVM0031351;EVM0024755;EVM0030416;EVM003	EVM0031351	0.22	0.43	NA	uncharacterized protein
	10	17147411	12.51	0.18	EVM0013195;EVM0014545;EVM0024782;EVM002	EVM0013195	NA	NA	NA	uncharacterized protein
	10	25222572	37.89	0.67	EVM0015298;EVM0001357;EVM0013490;EVM001	EVM0015812/Emp24	0.01	0.67	AT1G26690	emp24 family protein
	10	27149306	18.70	0.24	EVM0028266	EVM0028266	0.04	0.40	AT1G26170	importin-9 isoform X1
	10	29154724	31.25	0.51	EVM0009649;EVM0016152;EVM0010861	EVM0009649	0.19	-0.37	AT1G65730	probable
	11	3018112	13.73	0.32	EVM0011766;EVM0004195;EVM0031369;EVM001	EVM0011766	0.27	0.47	NA	dirigent protein 2-like
	11	19376565	43.05	1.08	EVM0007181;EVM0021826	EVM0011719	0.37	0.08	AT4G30870	crossover junction
	11	22243613	19.10	0.45	EVM0014349;EVM0023363;EVM0013728;EVM002	EVM0014349	0.14	0.27	AT2G28380	double-stranded
									NA	NA
	1	8347626	24.09	0.35	EVM0020113;EVM0033855;EVM0027956;EVM001	EVM0032114/KIX8	0.03	0.50	AT3G24150	seed development
	1	15839432	9.82	0.18	EVM0009771;EVM0001172;EVM0027661;EVM002	EVM0009771	0.14	0.29	NA	uncharacterized protein
	1	20948492	17.41	0.35	EVM0027453;EVM0001752;EVM0025906;EVM002	EVM0027453	0.13	-0.37	NA	RING-H2 finger protein
	1	41260863	38.99	0.17	NA	NA	NA	NA	NA	NA
	1	59463186	13.89	0.28	EVM0011025;EVM0029859;EVM0016896;EVM000	EVM0011025	0.43	-0.04	AT5G64380	fructose-1,6-bisphosphatase
	1	69664263	69.98	0.70	EVM0002363;EVM0009543;EVM0013498;EVM000	EVM0002363	0.19	0.67	AT1G64110	uncharacterized AAA
	2	12040684	12.83	0.24	EVM0020241;EVM0002777;EVM0025499;EVM001	EVM0020241	0.33	0.31	AT2G41830	uncharacterized protein
	2	53144136	22.06	0.49	NA	NA	NA	NA	NA	NA
	4	10069367	34.19	0.50	EVM0001100;EVM0007826;EVM0010433;EVM002	EVM0001100	0.09	-0.30	AT4G08500	mitogen-activated protein
	4	19559337	16.80	0.32	EVM0022984	EVM0022984/flo2	NA	NA	Os04g06451	seed development
SL	4	23324880	27.38	0.60	EVM0003687	EVM0003687	NA	NA	NA	uncharacterized protein
	4	36707584	43.60	1.11	NA	NA	NA	NA	NA	NA
	4	36876485	57.28	0.24	NA	NA	NA	NA	NA	NA
	4	44019674	7.56	0.14	NA	NA	NA	NA	NA	NA
	5	4423277	25.19	0.25	EVM0022236;EVM0032583;EVM0027307;EVM000	EVM0022236	NA	NA	NA	hypothetical protein
	5	25819986	24.40	0.51	EVM0028719	EVM0028719	0.47	0.04	AT5G60930	kinesin-like protein
	6	875387	52.50	0.85	EVM0014447;EVM0024620;EVM0022903;EVM002	EVM0014447	0.17	NA	NA	_
	6	3030969	28.04	0.62	NA	NA	NA	NA	NA	NA
	6	25299233	75.49	0.83	EVM0014591;EVM0008117;EVM0009754;EVM001	EVM0014591	0.09	NA	AT5G06600	ubiquitin carboxyl-terminal
	7	2931112	29.60	0.69	EVM0020569	EVM0020569	0.39	0.12	AT3G28910	transcription factor MYB30

	7	16448341	37.56	0.70	EVM0028878;EVM0001693;EVM0014504;EVM000	EVM0028878	0.10	0.43	NA	hypothetical protein
	7	18162529	40.71	0.32	NA	NA	NA	NA	NA	NA
	8	27254842	25.63	0.48	EVM0016552;EVM0007637;EVM0023167;EVM001	EVM0016552	0.29	0.39	NA	hypothetical protein
	8	35011031	38.52	0.97	EVM0031106;EVM0020703;EVM0011260;EVM002	EVM0031106	0.44	-0.04	AT5G04590	hypothetical protein
	8	35426274	13.18	0.23	EVM0002262;EVM0009943;EVM0010071;EVM001	EVM0002262	0.02	0.46	AT3G47450	NO-associated protein 1,
	9	4904833	65.69	0.70	EVM0002659;EVM0024225;EVM0033190;EVM002	EVM0002659	0.09	-2.15	AT1G05160	beta-amyrin 11-oxidase
	9	10979268	12.79	0.06	NA	NA	NA	NA	NA	NA
	9	24464422	64.19	1.81	EVM0000503	EVM0000503	0.16	-0.70	NA	hypothetical protein
	9	28397492	48.39	0.55	EVM0031454;EVM0020488;EVM0013581	EVM0031454	0.14	5.49	NA	ERBB-3 BINDING
	10	9340770	11.89	0.23	EVM0010402;EVM0005907;EVM0029863	EVM0010402	0.04	NA	NA	uncharacterized protein
	10	25223133	29.75	0.64	EVM0015298;EVM0001357;EVM0013490;EVM001	EVM0015812/Emp24	0.01	0.67	AT1G26690	emp24 family protein
	10	29162000	44.29	0.74	EVM0016152;EVM0019883;EVM0006214;EVM001	EVM0016152	0.13	-1.02	AT2G38290	ammonium transporter 3
	11	3999377	11.64	0.23	EVM0025961;EVM0003980;EVM0022286;EVM003	EVM0025961	0.01	1.41	NA	MLP-like protein 28
						NA	NA	NA	NA	NA
	1	26315874	39.79	1.58	EVM0011332;EVM0003799;EVM0029561;EVM001	EVM0011332	0.01	-1.16	NA	uncharacterized protein
	1	48794398	58.32	1.31	EVM0017136	EVM0017136	0.08	0.37	AT4G21060	hydroxyproline
	1	57873140	30.72	2.11	EVM0021938;EVM0010088	EVM0021938	NA	NA	NA	uncharacterized protein
	1	71147608	16.89	1.09	EVM0010900;EVM0004884;EVM0016378;EVM002	EVM0010900	0.05	1.90	NA	uncharacterized protein
	2	12602704	48.07	2.94	NA	NA	NA	NA	NA	NA
	2	41276874	16.20	0.54	EVM0012417;EVM0013452	EVM0012417	0.11	0.31	AT3G08960	importin-11 isoform X1
	2	52347869	17.66	0.58	NA	NA	NA	NA	NA	NA
SW	3	24593543	18.45	0.94	NA	NA	NA	NA	NA	NA
	3	38805325	28.32	2.08	NA	NA	NA	NA	NA	NA
	4	31168722	17.10	1.08	EVM0025782;EVM0025999;EVM0030918;EVM001	EVM0025782	0.07	0.34	AT2G36850	callose synthase 10 isoform
	4	36660846	15.53	0.99	EVM0005100;EVM0032848;EVM0019602;EVM000	EVM0005100	0.30	-0.14	NA	uncharacterized protein
	6	3200595	25.22	1.14	NA	NA	NA	NA	NA	NA
	6	13463604	27.54	1.62	EVM0001197;EVM0019910;EVM0006467;EVM001	EVM0028931/ZIP6	0.02	-0.85	AT2G30080	seed development
	7	4309297	23.26	0.59	EVM0008473;EVM0017648;EVM0012328	EVM0008473	0.02	-2.63	AT4G26190	uncharacterized FCP1
	7	17349239	15.69	0.33	EVM0018655	EVM0018655	0.39	-0.05	AT3G03960	hypothetical protein

10	4543462	34.77	1.37	EVM0030093;EVM0022792;EVM0012106;EVM000	EVM0030093	0.02	-0.41	NA	uncharacterized protein
10	25554659	13.80	0.64	EVM0012941;EVM0006124;EVM0023403	EVM0012941	0.07	0.22	AT5G60620	glycerol-3-phosphate
11	8044364	20.54	1.13	EVM0004020;EVM0027898;EVM0033076;EVM000	EVM0004020	0.04	1.39	AT1G78680	gamma-glutamyl hydrolase

§: The $_2$-values were calculated using paired *t*-test from the average RPKM values at three stages between two high seed weight (n_1=2) and tow seed weight (n_2=2) mungbeans, and their significances were marked by * (0.05 level); FC and NA represent fold change and no expression, respectively.

Supplemental Table 4. Thirteen significant QTNs commonly identified in two environments or two GWAS methods.

Trait	Genome-wide association studies					Comparative genomics			Arabidopsis homologs	Function
	Chr	Position (bp)	LOD score or P_1-value	r^2(%)	Method, Year	Candidate genes	P_2-value	log_2FC		
SL	1	35723799	43.02; 7.65E-09	1.39	3VmrMLM&EMMAX, 2018	EVM0015824	0.33	0.18	AT3G26590	DETOXIFICATION 29
SW	1	71543546	7.7~12.44	0.58~1.65	3VmrMLM, 2018, 2020	EVM0002784/BEE3	0.013	1.24	AT1G73830	seed development
HSW&SL	1	8161305-8347626	24.09~36.33	0.81~1.31	3VmrMLM, 2018, 2020	EVM0032114/KIX8	0.025	0.50	AT3G24150	seed development
HSW&SW	2	12602704	17.71~23.10	1.14~1.57	3VmrMLM, 2018, 2020	NA	NA	NA	NA	NA
HSW	4	47643044	17.25~18.0	0.46~1.33	3VmrMLM, 2018, 2020	EVM0033365	0.045	NA	AT4G36470	7-methylxanthosine synthase 1-like
HSW&SW	4	10069367	11.72~14.91	0.59~0.76	3VmrMLM, 2018, 2020	EVM0001100	0.087	-0.30	AT4G08500	mitogen-activated protein kinase kinase kinase 1-like
HSW&SL	4	22311675	11.98; 7.07E-12	0.42	3VmrMLM&EMMAX, 2018	EVM0006559	0.168	-1.158	NA	uncharacterized protein
HSW&SW	5	10834954	9.53~10.57	0.70~0.80	3VmrMLM, 2018, 2020	EVM0007856	0.126	0.24	AT2G38020	VACUOLELESS1 isoform X2
SL	6	20340225	2.38E-08		3VmrMLM&EMMAX, 2018	NA	NA	NA	NA	NA
SL&SW	8	2604294	16.58~24.71	1.41~1.73	3VmrMLM, 2018, 2020	EVM0016817	NA	NA	NA	NA
HSW&SW	9	5070156	7.80~20.77	0.47~1.45	3VmrMLM, 2018, 2020	EVM0029163	0.002	0.50	AT3G24870	EAF1 B isoform X7
HSW&SW&SL	10	25151160-25223155	15.40~37.89, 3.16E-08~5.15E-09	0.52-3.41	3VmrMLM&EMMAX&MLM	EVM0015812/Emp24	0.014	0.67	AT1G26690	emp24 family protein
SL	10	25369200	12.93~7.25E-07	0.98-1.70	3VmrMLM&MLM, 2018	EVM0013392	0.040	5.57	AT5G15310	transcription factor MYB106-like

The P_1-values were 3VmrMLM,The P_2-values were calculated using paired *t*-test from the average RPKM values at three stages between two high seed weight (n_1=2) and tow seed weight (n_2=2) mungbeans, and their significances were marked by * (0.05 level); FC and NA represent fold change and no expression, respectively.

Supplemental Table 5. Stable QTNs for seed-size-related traits in multiple environments.

Trait	Genome-wide association studies					Comparative genomics			Arabidopsis homologs	Function
	Chromosome	Position (bp)	LOD score or P_1-value	r^2(%)	Method, Year	Candidate genes	P_2-value	log_2FC		
HSW&SL	1	8161305-8347626	24.09~36.33	0.81~1.31	3VmrMLM, 2018, 2020	EVM0032114/KIX8	0.025	0.50	AT3G24150	seed development
HSW&SW	2	12602704	17.71~38.08	1.14~1.57	3VmrMLM, 2018, 2020	NA	NA	NA	NA	NA
HSW&SW	4	10069367	11.72~34.19	0.59~0.76	3VmrMLM, 2018, 2020	EVM0001100	0.087	-0.30	AT4G08500	mitogen-activated protein kinase kinase kinase 1-like
HSW&SW	5	10834954	9.53~30.03	0.70~0.80	3VmrMLM, 2018, 2020	EVM0007856	0.126	0.24	AT2G38020	VACUOLELESS1 isoform X2
HSW&SW &SL	10	25151160-25223155	15.40~37.89, 3.16E-08~5.15E-09	0.52-3.41	3VmrMLM&EMMAX&MLM	EVM0015812/Emp24	0.014	0.67	AT1G26690	emp24 family protein

Supplemental Table 6 The significant PPIs between key candidate genes identified in seed development pathways.

**Gene1**	**Arabidopsis homologs**	**Gene2 ID**	**Arabidopsis homologs**	**Predict score**
EVM0013794	AGG3/AT5G20635	EVM0006667	DA2/AT1G78420	0.478
EVM0013794	AGG3/AT5G20635	EVM0015092	GPA1/AT2G26300	0.577
EVM0033720	AGB/AT4G34460	EVM0013794	AGG3/AT5G20635	0.993
EVM0033720	AGB/AT4G34460	EVM0017944	GPA1-1/AT2G26300	0.995
EVM0033720	AGB/AT4G34460	EVM0015092	GPA1-2/AT2G26300	0.995

The critical score for protein-by-protein interaction was set at 0.40.

Supplemental Table 7 The significance of haplotypes in *VrEmp24/25* and *VrKIX8*

**Gene**	SNP				Cultivated mungbeans		Wild mungbeans		P-value
	No.	Genome region	NoH	EH	NoH	%	NO.	%	
Emp24/25	2	5'UTR	2	Hap1	160	90.9	9	45	2.11E-29
VrKIX8	6	CDS; UP	6	Hap1	129	73.3	8	40	1.19E-21

5'UTR: 5' untranslated region; UP: upstream; CDS: Coding sequence. NoH: No. of haplotypes; EH: elite haplotype. P-value is obtained from ANOVA for the traits of interest across various haplotypes.
